# Association of neovascular age-related macular degeneration with month and season of birth in Italy

**DOI:** 10.18632/aging.101137

**Published:** 2016-12-19

**Authors:** Antonio Longo, Alessandra Casuccio, Luca Pani, Teresio Avitabile, Salvatore Cillino, Maurizio G. Uva, Vincenza Bonfiglio, Andrea Russo, Guglielmo Parisi, Gilda Cennamo, Claudio Furino, Mariacristina Parravano, Entela Xoxi, Michele Reibaldi

**Affiliations:** ^1^ Azienda Policlinico-Vittorio Emanuele, Catania, Italy; ^2^ Departments of Sciences for Health Promotion and Mother Child Care, University of Palermo, Palermo, Italy; ^3^ Italian Medicines Agency, Rome, Italy; ^4^ Eye Clinic, University of Palermo, Palermo, Italy; ^5^ Eye Clinic, University of Naples Federico II, Naples, Italy; ^6^ Eye Clinic, University of Bari, Bari, Italy; ^7^ Fondazione G.B. Bietti, IRCCS, Rome, Italy

**Keywords:** neovascular age-related macular degeneration, neovascular AMD, anti-VEGF, season of birth, month of birth

## Abstract

In order to investigate the influence of season and month of birth on the risk of neovascular age-related macular degeneration (n-AMD) in Italy, we evaluated the month birth and sex of all patients, recorded in the anti-vascular endothelial growth factor (VEGF) monitoring registry of the Italian Medicines Agency, born between 1925–1944, who received intravitreal anti-VEGF injections for n-AMD between January 1, 2013 and July 29, 2015. The numbers of all births in Italy in the same years, extracted from the Italian National Institute of Statistics, were used to calculate the expected number of n-AMD cases. Overall, 45,845 patients (19,207 men, 26,638 women) received intravitreal anti-VEGF for n-AMD; in the same years, 20,140,426 people (10,334,262 male, 9,806,164 female) were born in Italy. Comparing the observed number of n-AMD cases with the expected number of n- AMD cases in each season, we found that the season-specific risk for n-AMD was 2.5% higher for those born in summer (OR=1.03, Bonferroni-corrected *P*=0.008) and 3% lower for those born in winter (OR=0.96, Bonferroni-corrected *P*=0.0004). When considering the month of birth, the risk of n-AMD was 5.9% lower for people born in January (OR=0.93, Bonferroni-corrected *P*=0.0012). The factors causing such differences should be determined.

## INTRODUCTION

Age-related macular degeneration (AMD), a progressive chronic disease of the central retina, is a major cause of blindness worldwide [1]. The prevalence of AMD is likely to increase as a consequence of the exponential increase in the aging population, and the projected number of people with AMD in 2040 is around 288 million [2]. Therefore, AMD will be a major medical and socioeconomic challenge worldwide in the coming years.

The precise pathogenesis is still poorly understood. It is generally accepted that AMD is the result of a complex interaction between genetic and environmental factors [3].

Several risk factors involved in the pathogenesis of AMD have been described, including genetic predisposition, age, and other modifiable factors such as smoking, light and ultraviolet (UV) exposure, dietary factors, and hypertension, which may influence the molecular mechanisms or cellular processes involved in development of the disease.

It is well established that the prevalence and incidence of many diseases are related to the month or season of birth [4-8]. There is increasing evidence that environmental factors in prenatal and early postnatal life can have significant effect on the development of various diseases later in adulthood. According to the “fetal origins hypothesis” of the developmental origins of the diseases, the susceptibility to many chronic diseases is determined in utero and has a lasting effect on the disease process [9].

No study has investigated whether there is an association between the season or month of birth and the risk of AMD. The aim of this study was to examine whether the month or season of birth is related to the risk of neovascular AMD (n-AMD) in Italy.

## RESULTS

Overall, in Italy, 60,818 patients received intravitreal injections of anti- vascular endothelial growth factor (VEGF) for n-AMD from January 1, 2013 to July 29, 2015. Consecutive years having at least 1000 individuals treated were found during the span 1925 to 1944 (included). A total of 46,826 patients born in these years were treated with anti-VEGF; after excluding 981 patients born outside Italy, a final sample of 45,845 patients (19,207 men and 26,638 women) who were born in Italy and treated with intravitreal anti-VEGF for n-AMD was obtained. In the same years, 20,140,426 people were born in Italy (10,334,262 males and 9,806,164 females) and were included in the analysis.

**Figure 1:**
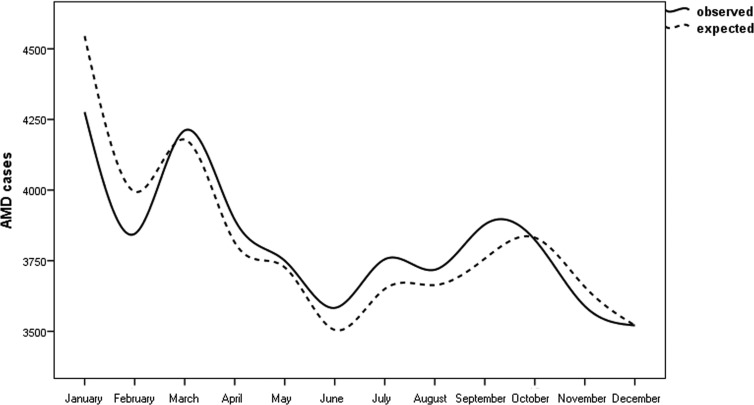
Pooled analysis of observed/expected births in people with neovascular AMD in Italy between 1925 and 1944 (n = 45845) with 95% confidence intervals

**Figure 2:**
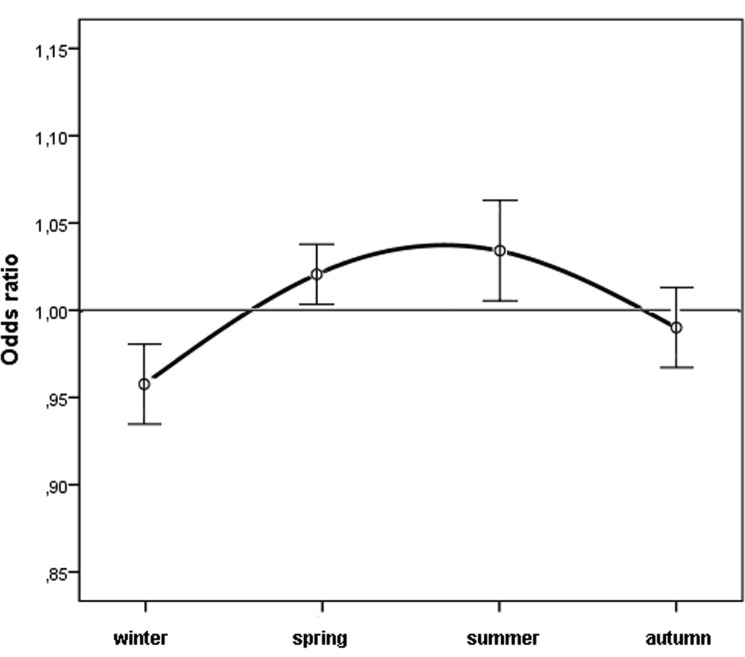
Odds ratios for people with neovascular AMD being born in different seasons in Italy between 1925 and 1944 (n = 45845) with 95% confidence intervals

Figure 1 shows the observed cases and the expected cases of n-AMD by month. In the analysis of the season-specific risk of n-AMD, we compared the number of people with n-AMD born in each season with that in the general population. The risk of n-AMD was 2.5% higher for people born in summer (OR = 1.03; [95% CI, 1.01–1.06]; χ^2^ = 3.057, *P* = 0.002; Bonferroni corrected *P* = 0.008) and 3% lower for those born in winter (OR = 0.96 ([95% CI, 0.94–0.98]; χ^2^ 4.03, *P* = 0.0001; Bonferroni corrected *P* = 0.0004) (Table 1 and Fig. 2).

**Table 1:** All borns, observed number of people with neovascular AMD compared with the expected number, odds ratio with 95% CI, according to season

	All borns			Observed n-AMD cases			Expected n-AMD cases			OR, 95 CI, p		
	male	female	all	male	female	all	male	female	all	male	female	all
Winter	2889994	2697229	5587223	5245	7087	12332	5371,3	7326,9	12718,0	**0.967; 0.94-0.99; p=0.042 ^1^**	**0.955; 0.93-0.98; p=0.001 ^3^**	**0.958; 0.94-0.98; p=0.0001 ^5^**
Spring	2490013	2362684	4852697	4756	6472	11228	4627,9	6418,1	11046,0	**1.037; 1.01-1.07; p=0.030 ^2^**	1.011; 0.98-1.04; p=0.439	**1.022; 1.01-1.04; p=0.047 ^6^**
Summer	2498596	2365135	4863731	4739	6612	11351	4643,8	6424,8	11071,2	1.027; 0.99-1.06; 0.108	**1.039; 1.011-1.069; p=0.006 ^4^**	**1.034; 1.01-1.06; p=0.002 ^7^**
Autumn	2455659	2381116	4836775	4467	6467	10934	4564,0	6468,2	11009,8	0.972; 0.94-1.01; p=0.099	1.0; 0.97-1.03; p=0.986	0.990; 0.97-1.01; p=0.407
Total	10334262	9806164	20140426	19207	26638	45845	19207	26638	45845			
p (Bonferroni correction)										**^1^** p=0.168	**^3^ p=0.004**	**^5^ p=0.0004**
										**^2^** p=0.120	**^4^ p=0.024**	**^6^** p=0.188
												**^7^ p=0.008**

**Figure 3:**
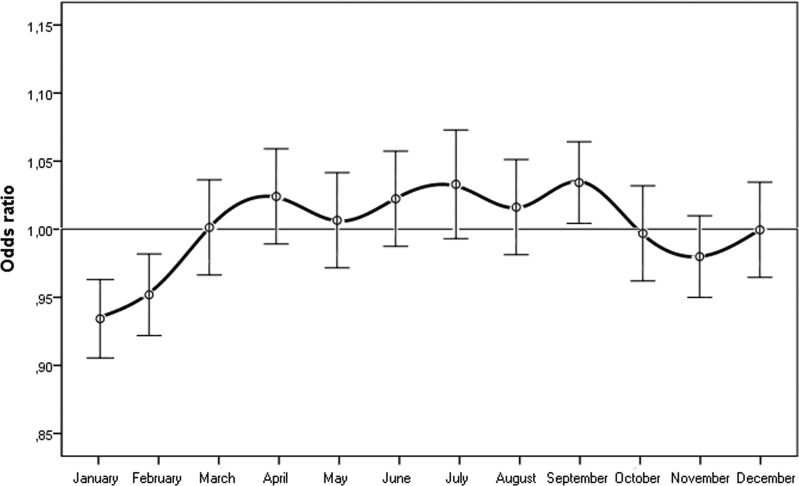
Odds ratios for people with neovascular AMD being born in different months in Italy between 1925 and 1944 (n = 45845) with 95% confidence intervals

**Figure 4:**
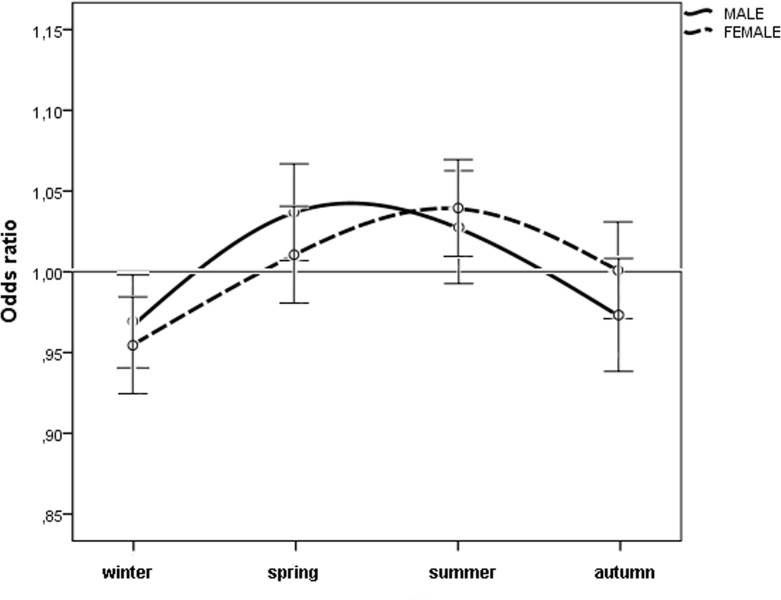
Odds ratios for men and women with neovascular AMD being born in different seasons in Italy between 1925 and 1944 (n = 45845) with 95% confidence intervals

**Figure 5:**
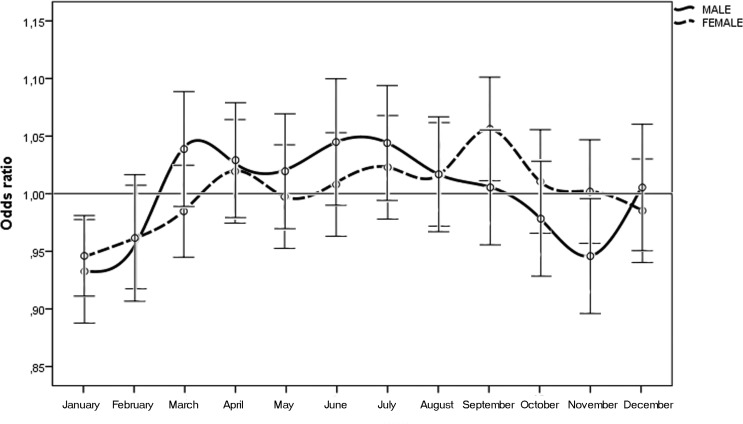
Odds ratios for men and women with neovascular AMD being born in different months in Italy between 1925 and 1944 (n = 45845) with 95% confidence intervals

When we considered the month of birth, the risk of n-AMD was 5.9% lower for people born in January (OR = 0.93 [95% CI, 0.91–0.96); χ^2^ = 4.20, *P* = 0.0001, Bonferroni corrected *P* = 0.0012) (Table 2 and Fig. 3). The results of the subgroup analysis are shown in Tables 1 and 2, and Figures 4 and 5. In women, the risk of n-AMD was 2.9% higher for those born in summer (OR = 1.04 [95% CI, 1.01–1.07); χ^2^ = 2.68, *P* = 0.006, Bonferroni corrected *P* = 0.024) and 3.2% lower for those born in winter (OR = 0.95 [95% CI, 0.93–0.98); χ^2^ = 3.29, *P* = 0.001, Bonferroni corrected *P* = 0.004). In women, no significant differences were found between different months after Bonferroni correction. In men, no significant differences were found between seasons or months.

**Table 2:** All borns, observed number of people with neovascular AMD (n-AMD) compared with the expected number, odds ratio with 95% CI, according to month

	All borns			Observed n-AMD cases			Expected n-AMD cases			OR, 95 CI, p		
	male	female	all	male	female	all	male	female	all	male	female	all
January	1052187	944371	1996558	1838	2438	4276	1955,6	2565,3	4544,7	**0.933; 0.89-0.98; p=0.005 ^1^**	**0.945; 0.91-0.98; p=0.008 ^3^**	**0.935; 0.91-0.96; p=0.0001 ^5^**
February	897659	857274	1754933	1598	2247	3845	1668,4	2328,7	3994,7	0.954; 0.90-1.01; p=0.070	0.961; 0.92-1.01; p=0.075	**0.959; 0.93-0.99; p=0.013 ^6^**
March	940148	895584	1835732	1809	2402	4211	1747,3	2432,8	4178,6	1.041; 0.99-1.09; P=0.121	0.986; 0.95-1.03; p=0.512	1.008; 0.97-1.04; p=0.599
April	858981	816864	1675845	1634	2261	3895	1596,5	2219,0	3814,7	1.026; 0.98-1.08; p=0.326	1.020; 0.98-1.07; p=0.351	1.023; 0.99-1.06; p=0.174
May	839404	797651	1637055	1590	2160	3750	1560,1	2166,8	3726,4	1.021; 0.97-1.07; p=0.429	0.997; 0.95-1.04; p=0.879	1.006; 0.97-1.04; p=0.686
june	791628	748169	1539797	1532	2051	3583	1471,3	2032,4	3505,0	1.045; 0.99-1.10; p=0.099	1.010; 0.97-1.06; p=0.667	1.024; 0.99-1.06; p=0.169
July	823977	779541	1603518	1593	2162	3755	1531,4	2117,6	3650,0	1.044; 0.99-1.010; p=0.101	1.023; 0.98-1.07; p=0.314	1.031; 0.99-1.07; p=0.069
August	826973	782608	1609581	1561	2157	3718	1537,0	2125,9	3663,8	1.017; 0.97-1.07; p=0.523	1.016; 0.97-1.06; p=0.482	1.016; 0.98-1.05; p=0.350
September	847646	802986	1650632	1585	2293	3878	1575,4	2181,3	3757,3	1.006; 0.96-1.06; p=0.800	**1.056; 1.01-1.10; p=0.012 ^4^**	**1.035; 1.01-1.07; p=0.039 ^7^**
October	863423	819891	1683314	1575	2249	3824	1604,7	2227,2	3831,7	0.979; 0.93-1.03; p=0.438	1.011; 0.97-1.06; p=0.629	0.998; 0.96-1.03; p=0.897
November	821012	785681	1606693	1451	2138	3589	1525,9	2134,3	3657,3	**0.946; 0.89-0.99; p=0.045 ^2^**	1.002; 0.96-1.05; p=0.933	0.980; 0.95-1.01; p=0.239
December	771224	775544	1546768	1441	2080	3521	1433,4	2106,7	3520,9	1.005; 0.95-1.06; p=0.834	0.986; 0.94-1.03; p=0.543	1.0; 0.97-1.04; p=0.998
total	10334262	9806164	20140426	19207	26638	45845	19207	26638	45845			
p (Bonferroni correction)										**^1^** p=0.06	**^3^** p=0.096	**^5^ p=0.0012**
										**^2^** p=0.54	**^4^** p=0.144	**^6^** p=0.156
												**^7^** p=0.468

## DISCUSSION

In this study, we analyzed the month of birth in the years 1925 to 1944 of patients with n- AMD who were treated with intravitreal anti-VEGF injections in Italy. We found a seasonal variation: the number of people who developed n-AMD was higher than expected in summer, and lower in winter.

Several diseases exhibit an effect of birth month or birth season on the incidence.[4-8] A meta-analysis of studies on multiple sclerosis showed a higher risk in patients born in April and May and lower risk in those born in October and November, as well as a relationship between risk and latitude [6]. Similar trends have been reported for other immune-mediated diseases. A large study of people with type 1 diabetes in the USA found that the relative percentage of observed to expected births was lower in November–February and higher in April–July. This effect was seen in both men and women but was evident only in regions in the northern latitudes, which suggested a role of factors related to geographic latitude such as solar irradiance [5].

A national study in the UK showed that the distribution of births of patients who developed an immune-mediated disease (e.g., multiple sclerosis, rheumatoid arthritis, ulcerative colitis, or systemic lupus erythematosus) differed significantly from that of the general population. The peak of births for those who developed one of these diseases occurred in April and a trough in October, and the risk correlated inversely with predicted second-trimester UVB exposure and third-trimester vitamin D status [7].

Several seasonal factors acting during pregnancy and the perinatal period have been proposed to explain these data, including infections, dietary factors (e.g., low intake of antioxidants in winter), and vitamin D deficiency through lack of sun exposure during pregnancy.[8]. This latter plays a significant role, since increased serum vitamin D concentration is associated with a decreased risk of multiple sclerosis [10,11], and vitamin D supplementation during the first year of life is associated with a significantly decreased risk of type 1 diabetes [12].

The biologically active form of vitamin D is generated in the skin during exposure to UV radiation from sunlight, and its levels follow a seasonal distribution. In the Unites States, a study of more than 3 million people reported a peak of 25-hydroxyvitamin D (25(OH)D) level in August and lowest level in February [13]. In young women in Italy, the serum concentration in summer was reported to be twice that in winter (120 nmol/l in July vs 60 nmol/l in February) [14]. During pregnancy, when a higher amount of vitamin D is required, many pregnant women can develop a vitamin D deficiency. In Ireland, a higher prevalence of vitamin D deficiency (25(OH)D <27.5 nmol/l) was observed in pregnant than in nonpregnant women, both in spring (44% vs. 18%) and in winter (35% vs 29%) [15]. Newborns can have lower vitamin D levels than their mothers. In the USA, in winter and spring, a vitamin D deficiency (25(OH)D <37 nmol/l) was found in 5% of new mothers and in 9.7% of their newborns [16]. In Italy, low vitamin D levels (25(OH)D<25 nmol/l) were found in 18% of new mothers and in 38% of their newborns [17].

Gestational vitamin D concentration is considered important for the development of the immune system. The vitamin D acts as an immunomodulator by inhibiting the proliferation of the T cells, shifting the T-cell population toward noninflammatory T helper 2 (Th2) and T-regulatory cells, and suppressing the transcription of genes encoding key Th1 pro-inflammatory cytokines [18]. Genes associated with many autoimmune diseases are enriched for vitamin D receptor-binding sites [19-21], suggesting that the combined effect of vitamin D deficiency, genetic variation, and exposure to other environmental agents may concur in the pathogenesis. Seasonal changes in immune function are also mediated by the pineal hormone melatonin, which has a proinflammatory action [31], by increasing the release of Th1 cytokines [22].

In animal models, gestational vitamin D deficiency affects brain development [23], and the myelination process [24], and it could have an effect on retinal cells. Vitamin D is involved in the pathogenesis of neovascular AMD, and a significant correlation has been found between reduced plasma vitamin D level and the prevalence of AMD [25-28]. A recent meta-analysis reported a correlation between latitude, insolation, and the incidence of severe AMD; higher incidence was found in locations with insolation <3 kWh/m^2^/day compared with those with insolation >3 kWh/m^2^/day (*P* < 0.001) [29].

An effect of the month of birth has also been reported for ocular conditions such as myopia. In Israel, a study found increased rates of moderate and severe myopia in people born in summer and lower rates in those born in winter (9% and 8.5%, and 2.2% and 2.7%, respectively). These differences correlated with the number of daylight hours, which is thought to relate to the melatonin level, or an imbalance in the melatonin–dopamine relationship.[30]. Similarly, a study in the UK reported a 4.1% increased rate of severe myopia in summer–autumn and 3.6% lower rate in winter–spring. However, the lack of relationship between severe myopia and the number of daylight hours, suggested the influence of other season-related factors, such as the birth weight [31].

Studies of the effect of birth month or birth season have observed significant variability in birth month in the general population. This factor, combined with the inevitable heterogeneity in region of origin and year of birth, can lead to false-positive associations [32]. In this study, we included people born throughout Italy over a 20-year period (1925–1944), thus avoiding the possible effect of a single year.

This study has several limitations. Although season of birth is a well-defined variable, it is only one possible factor in the pathogenesis of the n-AMD. It is associated with various other environmental factors, such as meteorological factors, daily sunlight exposure, and alterations in air pollution and food supply, as well as behavioral variables, including dietary habits and physical activity levels. Personal factors, such as smoking, urban or rural residency, alimentary habits, and migration (inside or outside Italy) could have caused different levels of exposure to risk factors and had an effect on the development of n-AMD.

In conclusion, this study shows that there is seasonal variation in the risk of developing neovascular AMD in Italy. The factors causing such changes should be determined.

## METHODS

In this study, all consecutive patients recorded in the anti-VEGF monitoring registry of the Italian Medicines Agency (AIFA) who received intravitreal injections of anti-VEGF for n-AMD between January 1, 2013 and July 29, 2015 were included. The system monitors the registration process throughout the country and uploading of clinical data, and allows access to data about reimbursement by the National Health Service [33,34].

The inclusion criteria were patients born in Italy and treated with intravitreal anti-VEGF injection for n-AMD. Patients born outside Italy were excluded. The data obtained from the AIFA database for each patient were year and month of birth, region of birth, and sex. Other parameters allowing personal identification (such as city or date of birth, or initials of patients) were not provided. The drugs used for intravitreal injections of anti-VEGF recorded in the AIFA registry included in this study were pegaptanib sodium (Macugen; EyetechInc, Palm Beach Gardens, FL), bevacizumab (Avastin; Genentech Inc., South San Francisco, CA), ranibizumab (Lucentis; Genentech Inc.), and aflibercept (EYLEA; Regeneron Pharmaceuticals, Inc, Tarrytown, NY).

From the initial cohort of patients, to ensure a sufficient sample size across all 12 months, as previously described [4], we included in the analysis only those consecutive years having at least 1000 individuals treated. For the same years, the numbers of all births in Italy were extracted from the Italian National Institute of Statistics along with information about the month of birth, sex, and region of birth. These data were used to calculate the expected number of births of people who would later develop AMD under the assumption that the birth distributions for patients and the whole population are equal.

Institutional review board approval was obtained.

### Statistical analysis

The n-AMD incidence rate was calculated per 100,000 residents per year. The distribution of season of birth was compared between the study population (with n-AMD) and the corresponding Italian population. We compared the observed number of n- AMD cases with the expected number of n-AMD cases in each season, and we analyzed the season-specific risk of n-AMD compared with the other 3 seasons using the odds ratios (ORs) and 95% confidence intervals (CIs) and a 2 × 2 chi-square test. Significant (P ≤ 0.05) values for a single season were corrected for the 4 comparisons using the Bonferroni correction.

We also compared the observed number of n-AMD cases with the expected number of n-AMD cases in each month, and we analyzed the month-specific risk of n-AMD compared with the other 11 months using ORs and 95% CIs and a 2 × 2 chi-square test. Significant (P ≤ 0.05) values for single months were corrected for the 12 comparisons using the Bonferroni correction.

Subgroup analyses were performed for women and men. We calculated 95% CIs based on the Poisson distribution for describing the variation in the number of events during a period of time.

Data were analyzed using IBM SPSS Software 22 version (IBM Corp., Armonk, NY, USA). All P-values were 2-sided, and P ≤ 0.05 was considered to be significant.
